# Interfacial Distortion of Sb_2_Te_3_–Sb_2_Se_3_ Multilayers via Atomic Layer Deposition for Enhanced Thermoelectric Properties

**DOI:** 10.1021/acsnano.3c13152

**Published:** 2024-06-26

**Authors:** Jun Yang, Mohammadreza Daqiqshirazi, Tobias Ritschel, Amin Bahrami, Sebastian Lehmann, Daniel Wolf, Wen Feng, Almut Pöhl, Jaroslav Charvot, Filip Bureš, Thomas Brumme, Axel Lubk, Jochen Geck, Kornelius Nielsch

**Affiliations:** † Leibniz Institute for Solid State and Materials Research, Dresden 01069, Germany; ‡ Institute of Materials Science, Technische Universität Dresden, Dresden 01062, Germany; § Chair of Theoretical Chemistry, Technische Universität Dresden, Dresden 01069, Germany; ∥ Institute of Solid State and Materials Physics, Technische Universität Dresden, Dresden 01069, Germany; ⊥ Institute of Organic Chemistry and Technology, Faculty of Chemical Technology, University of Pardubice, Pardubice 53210, Czech Republic

**Keywords:** atomic layer deposition, Sb_2_Te_3_−Sb_2_Se_3_, 2D materials, interface engineering, transport property

## Abstract

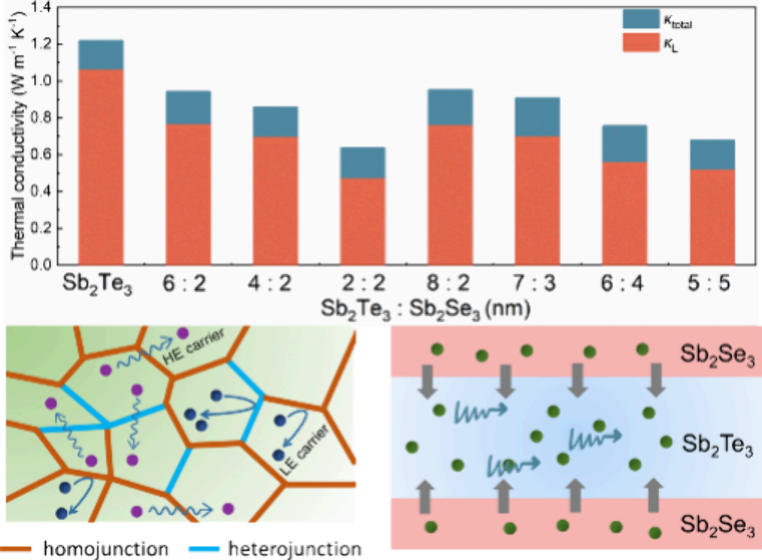

Atomic layer deposition (ALD) is an effective technique for depositing thin films with precise control of layer thickness and functional properties. In this work, Sb_2_Te_3_–Sb_2_Se_3_ nanostructures were synthesized using thermal ALD. A decrease in the Sb_2_Te_3_ layer thickness led to the emergence of distinct peaks from the Laue rings, indicative of a highly textured film structure with optimized crystallinity. Density functional theory simulations revealed that carrier redistribution occurs at the interface to establish charge equilibrium. By carefully optimizing the layer thicknesses, we achieved an obvious enhancement in the Seebeck coefficient, reaching a peak figure of merit (*zT*) value of 0.38 at room temperature. These investigations not only provide strong evidence for the potential of ALD manipulation to improve the electrical performance of metal chalcogenides but also offer valuable insights into achieving high performance in two-dimensional materials.

## 1 Introduction

Thermoelectric (TE) materials have the capacity to directly convert heat energy into electrical power, rendering them highly promising for microenergy harvesting applications in Internet of Things devices.^1^ The heat-to-electricity conversion efficiency of thermoelectric materials can be evaluated by the dimensionless figure of merit (*zT*), which is defined as *zT* = σ*S*^2^*T*/κ, where σ, *S*, *T*, and κ are the electrical conductivity, Seebeck coefficient, absolute temperature, and total thermal conductivity, respectively. κ is a combination of electronic (κ_E_) and lattice (κ_L_) thermal conductivities. Enhancing the TE power factor (σ*S*^2^) in any material necessitates precise control of the critical transport parameters. A high power factor in thermoelectric materials indicates their efficient conversion of a temperature difference into electrical power.^2^ This efficiency enhancement results in increased power output, enhanced energy conversion efficiency, reduced energy losses, and optimal power transfer, making high power factor thermoelectric materials highly sought after for various applications. However, these thermoelectric parameters are intrinsically intertwined, posing a significant hurdle in their independent optimization.

One of the effective strategies to enhance thermoelectric performance is to reduce the dimensions, thereby introducing the quantum confinement effect in nanostructured thermoelectric materials.^3^ The electronic states are confined by closed geometries at the nanometer scale, leading to quantized energy levels and enhancing the density of electron states per unit volume occurring for a small well within.^4^ In this case, the carrier effective mass and Seebeck coefficient can be elevated rapidly. Hicks et al. demonstrated a 3-fold increase in the Seebeck coefficient value of PbTe/Pb_0.927_Eu_0.073_Te compared to bulk PbTe, attributed to the quantum confinement effect in the nanostructured system.^5^ As an alternative, Gao et al. fabricated PbSe/SnSe multiple quantum well structures, achieving a high power factor of 25.7 μW cm^–1^ K^–2^ at 300 K, which is 4 times larger than that of a PbSe single layer.^6^ Mune et al. implemented a SrTi_0.8_Nb_0.2_O_3_ (conductor) layer between SrTiO_3_ (insulator) films, resulting in an improved Seebeck coefficient from 61 to 320 μV K^–1^.^7^ However, as the insulator barrier does not contribute to electronic transportation, there was no improvement in the carrier concentration and electrical conductivity.

In addition to high *S*, κ is also a crucial parameter in determining the thermoelectric performance of a material. Ren and Dow demonstrated that the thermal conductivities of multilayer thin films are lower than those of their corresponding bulk materials, employing the classical Boltzmann transport equation approach with a quantum mechanical treatment of the scattering rate.^8^ The presence of high-density superlattice interfaces leads to strong phonon scattering, resulting in a significant reduction in lattice thermal conductivity. The Bi_2_Te_3_ (1 nm)/Sb_2_Te_3_ (5 nm) superlattice structure constructed by Winkler yielded an ultralow lattice thermal conductivity of 0.23 W m^–1^ K^–1^.^9^ Tang and colleagues recently fabricated the MoTe_2_/Bi_2_Te_3_ system. The interfacial scattering of phonons has resulted in a notable reduction of κ_L_, with values in the produced superlattice films ranging from 0.31 to 0.39 W m^–1^ K^–1^.^10^ In addition, several factors that contribute to reducing thermal conductivity, including lattice mismatch at the interfaces, alloy scattering, phonon minigap formation, and phonon tunneling, have been investigated with the aim of optimizing thermal conductivity in superlattices.^11−13^

While artificially engineered thermoelectric nanostructures hold great promise for synergistically optimizing transport parameters, the rational design, reliable fabrication, and the mechanism responsible for optimizing electrical properties remain elusive and pose significant challenges. Here, nanostructured thin films of Sb_2_Te_3_–Sb_2_Se_3_ were successfully synthesized using thermal ALD,^14^ with the composition of this nanostructure precisely controlled by adjusting the ALD cycle numbers for Sb_2_Te_3_ and Sb_2_Se_3_. A systematic study of the crystal structures and transport properties revealed an obvious improvement in the thermoelectric performance upon careful optimization of the Sb_2_Se_3_ sublayer thickness. The sample Sb_2_Te_3_:Sb_2_Se_3_ = 5:5 (nm) exhibited an impressive Seebeck coefficient of 174 μV K^–1^ at room temperature. Further investigation demonstrated a high power factor of 852 μW m^–1^ K^–2^ accompanied by a peak *zT* value of 0.38 at room temperature.

## 2 Results and Discussion

**Figure 1:**
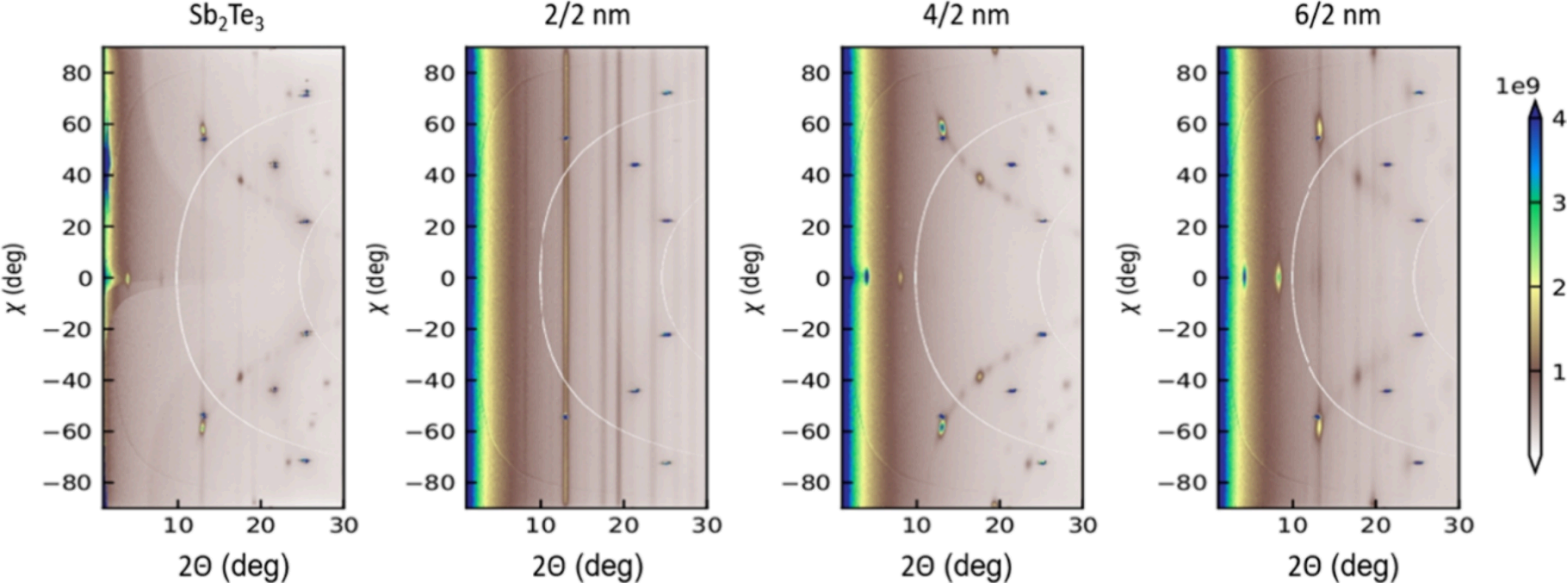
Regrouped scattered X-ray intensity as a function of scattering angle 2θ and polar angle χ, i.e., angle along the Laue cone for the samples Sb_2_Te_3_ and with Sb_2_Te_3_:Sb_2_Se_3_ ratios of 2:2, 4:2, and 6:2 (nm).

Before the structures were synthesized, the growth behaviors of single-phase Sb_2_Te_3_ and Sb_2_Se_3_ films were investigated. For Sb_2_Te_3_ films, a combination of SbCl_3_ and (Et_3_Si)_2_Te precursors was employed, whereas for Sb_2_Se_3_ films, SbCl_3_ and Se(SnMe_3_)_2_ precursors were utilized.^15,16^ The Sb_2_Te_3_ and Sb_2_Se_3_ thin films were grown at 80 and 110 °C without any vacuum break, respectively (see more details in the Experimental Section). The structural characterizations of Sb_2_Te_3_–Sb_2_Se_3_ are shown in Figure 1 and Figure S1 in the Supporting Information. Grazing incidence X-ray diffraction (GID) measurements were performed using a custom-built laboratory setup equipped with a Mo Kα source to analyze the structural characteristics of the samples. The corresponding data reveals that Sb_2_Te_3_ exhibits a rhombohedral phase with a space group of *R*3̅*m*, while Sb_2_Se_3_ adopts an orthorhombic phase with a *Pbnm* space group. As the thickness of the Sb_2_Te_3_ layer decreases, distinctive peaks emerge from the Laue rings, signifying a highly textured film structure achieving optimal crystallinity in the sample Sb_2_Te_3_:Sb_2_Se_3_ = 4:2 (nm). On the other hand, the sample Sb_2_Te_3_:Sb_2_Se_3_ = 2:2 (nm) has a lower diffraction intensity than the other samples, suggesting a smaller grain size.

**Figure 2:**
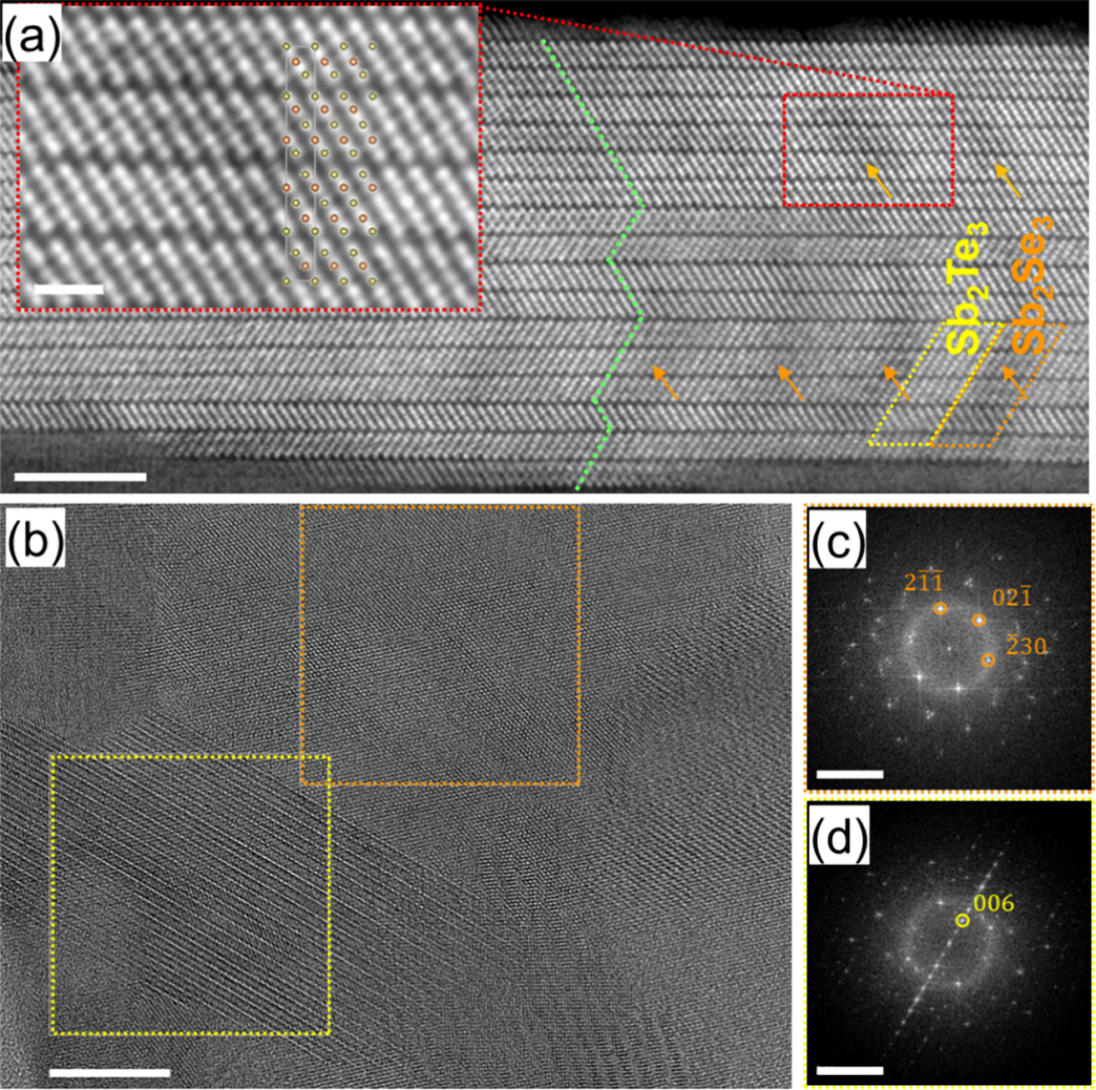
Structural characterizations of Sb_2_Te_3_–Sb_2_Se_3_ thin films. (a) HAADF-STEM image of a Sb_2_Te_3_–Sb_2_Se_3_ multilayer grain taken along its [010] zone-axis orientation and *c* axis in the vertical direction (scale bar: 5 nm). The inset shows a zoom-in of the region marked by the red dashed box, in which the atomic Sb_2_Te_3_ model is overlaid (scale bar: 1 nm). The orange arrows highlight the Sb_2_Se_3_ regions, which appear slightly darker in the image due to their lower Z-contrast. (b) HRTEM images show different grains of the poly crystalline structure (scale bar: 10 nm). (c, d) Fourier transforms (FTs) of regions marked by orange (c) and yellow (d) dashed boxes in panel (b) (scale bars: 5 nm^–1^). (c) FT shows reflections corresponding to the [324] orientation of the orthorhombic phase with a *Pbnm* space group. (d) The FT shows, e.g., the 006 reflection, indicating a direction parallel to the *c* axis of the *R*3̅*m* space group.

To investigate the microstructure of the Sb_2_Te_3_–Sb_2_Se_3_ nanocomposites, cross-sectional imaging at atomic resolution was performed using high-resolution transmission electron microscopy in both bright-field mode (HRTEM) and high-angle annular dark-field scanning mode (HAADF-STEM). A representative HAADF-STEM image of a single grain taken along [010] zone-axis orientation according to the rhombohedral Sb_2_Te_3_ crystal structure is shown in Figure 2a. The latter reveals an atomic zigzag arrangement between the mono-, bi-, and multilayers highlighted by a green dashed line indicating the existence of twin boundaries.^17^ Furthermore, a faint bright-dark atomic (Z-) contrast modulation is visible with a periodicity in the horizontal direction of about 2 nm in which the Te atoms appear brighter (*Z* = 52) than the Se atoms (*Z* = 34). In fact, this coincides with the intended alternation between Sb_2_Te_3_ and Sb_2_Se_3_ deposition, where in this case the Sb_2_Se_3_ adopts the *R*3̅*m* crystal structure of the Sb_2_Te_3_. This observation is also confirmed by STEM energy-dispersive X-ray mapping (STEM-EDX), as illustrated in Figure S2 of the Supporting Information. It should be noted that ALD growth at relatively low temperatures leads to polycrystalline layers. For low-cycle ALD of Sb_2_Te_3_, the presence of dangling bonds at the layer edges preferentially adsorbs precursor molecules.^18^ This phenomenon may diverge from the conventional monolayer-by-monolayer arrangement, instead leading to the formation of an expanding alloy cluster (Volmer–Weber growth behavior).^18,19^ Furthermore, the exchange reaction at the gas–solid interface during the ALD can contribute to alloying. The exposure of the Se precursor onto the Sb_2_Te_3_ surface leads to the exchange of Se by Te atoms at the interface, resulting in the formation of alloy clusters and atomic-scale distortions.^20^ For example, the HRTEM image in Figure 2b depicts different grains, two of which are marked with dashed squares in yellow and orange. The corresponding Fourier transforms (FTs) of these marked regions displayed in Figure 2c,d are consistent with two different structures. The FT of the upper grain (Figure 2c) shows reflections corresponding to the [324] orientation of the orthorhombic phase with a *Pbnm* space group, whereas the FT of the lower grain (Figure 2d) reveals, e.g., the 006 reflection, indicating a direction parallel to the *c* axis of the *R*3̅*m* space group. The incorporation of dopants into the pristine layered films has been observed to improve the Seebeck coefficient, which will be discussed in the following sections.

**Figure 3:**
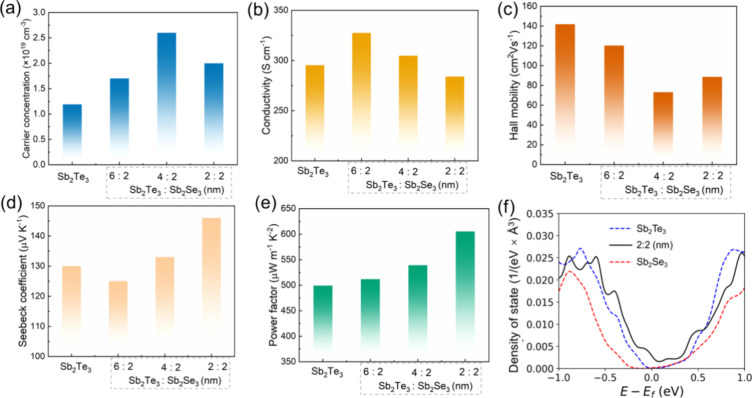
Transport properties of Sb_2_Te_3_–Sb_2_Se_3_ nanostructured films: (a) carrier concentration, (b) electrical conductivity, (c) mobility, and (d) Seebeck coefficient. (e) Power factor of thin films. (f) Density of states (DOS) for the three bulk systems of Sb_2_Te_3_, Sb_2_Se_3_, and Sb_2_Te_3_–Sb_2_Se_3_ films.

Figure 3 depicts the room temperature electrical transport properties of the Sb_2_Te_3_–Sb_2_Se_3_ structures. The carrier concentration of the Sb_2_Te_3_ thin film is 1.18 × 10^19^ cm^–3^ and reaches a peak value of 2.61 × 10^19^ cm^–3^ in the Sb_2_Te_3_:Sb_2_Se_3_ = 4:2 (nm) structure. The carrier concentration of Sb_2_Se_3_ is 3.6 × 10^18^ cm^–3^ at room temperature. Considering the bandgaps for Sb_2_Te_3_ and Sb_2_Se_3_ as 0.3 and 1.14 eV, respectively, a band offset occurs for the Sb_2_Te_3_–Sb_2_Se_3_ heterostructure.^21−23^ The significantly higher work function difference between Sb_2_Se_3_ and Sb_2_Te_3_ results in a pronounced hole junction from Sb_2_Se_3_ to Sb_2_Te_3_.^6,24^ The thicker Sb_2_Te_3_ layer in the structure facilitates a larger potential drop at the Sb_2_Te_3_/Sb_2_Se_3_ interface, leading to a higher carrier concentration compared to a thinner Sb_2_Te_3_ layer.^6^ However, a slight decrease in the carrier concentration is observed when the Sb_2_Te_3_ to Sb_2_Se_3_ ratio is further reduced. Specifically, the carrier concentration of the Sb_2_Te_3_:Sb_2_Se_3_ = 2:2 (nm) sample is lower than that of Sb_2_Te_3_:Sb_2_Se_3_ = 4:2 (nm). In the Sb_2_Te_3_:Sb_2_Se_3_ = 2:2 (nm) sample, the Sb_2_Te_3_ layer is too thin to effectively shield the built-in potential, resulting in carrier pinning at the interface.^6^ Under these conditions, charge transfer occurs more readily in Sb_2_Se_3_ with a thicker Sb_2_Te_3_ layer, leading to a higher carrier concentration. The highest electrical conductivity of 327 S cm^–1^ was achieved at Sb_2_Te_3_:Sb_2_Se_3_ = 6:2 (nm) (Figure 3b). The Hall mobility of the systems falls within the range of 90–120 cm^2^ V^–1^ s^–1^, which is lower than a value of 140 cm^2^ V^–1^ s^–1^ observed in the Sb_2_Te_3_ layer (Figure 3c). Interface scattering arises when carriers traverse between different layers, leading to mobility restrictions and impacting overall mobility.^25^ Additionally, potential barriers within the superlattice structure can obstruct the interlayer transport of charge carriers, further reducing mobility.^26^ Furthermore, interface effects can induce the formation of interface states, which act as scattering centers for carriers, contributing to additional charge scattering and a subsequent decrease in mobility.^27^ Conversely, an inverse trend is evident in the Seebeck coefficient results. For instance, as depicted in Figure 3d, the lowest Seebeck coefficient of 125 μV K^–1^ is obtained at Sb_2_Te_3_:Sb_2_Se_3_ = 6:2 (nm), while the Sb_2_Te_3_:Sb_2_Se_3_ = 2:2 (nm) sample exhibits the highest value of 146 μV K^–1^. The room temperature transport properties correspond well with the theoretical calculation results based on replacement modes, which can be supported by density functional theory results in Table S2 and Figures S3 and S4 in the Supporting Information. The bandgap is minimally affected by the strain induced by the lattice mismatch between Sb_2_Te_3_ and Sb_2_Se_3_. Instead, it is the states far from the Fermi energy that are primarily influenced. The strain can alter the thermoelectric coefficient slightly due to the changes in the electronic states. Further discussion on lattice mismatch can be found in the Supporting Information (Table S3 and Figure S5).

The single parabolic band (SPB) model is the most widely employed approach for evaluating the electrical transport properties of thermoelectric materials.^28,29^ The relationship between the Seebeck coefficient and carrier concentration at room temperature was determined using the Pisarenko plot, as shown in Figure S6 in the Supporting Information. The effective mass (m*) increases from 0.38m_e_ to 0.65m_e_ with the rising doping level of Sb_2_Se_3_ into the Sb_2_Te_3_ system. Notably, the m* of the Sb_2_Te_3_–Sb_2_Se_3_ layer is significantly higher than that of the Sb_2_Te_3_ layer, indicating the presence of band bending and the energy filtering effect in the heterostructure system. Additionally, the incorporation of the quantum confinement effect in the multilayer system further enhances m*, given that the thickness is smaller than the de Broglie wavelength of electrons.^30^ The power factor was calculated using the electrical conductivity and Seebeck coefficient data (Figure 3e). A maximum PF value of 605 μW m^–1^ K^–2^ was determined for the sample Sb_2_Te_3_:Sb_2_Se_3_ = 2:2 (nm) at room temperature, primarily attributed to the high Seebeck coefficient. The sample Sb_2_Te_3_:Sb_2_Se_3_ = 2:2 (nm) appears more semimetallic, as indicated by the DOS not approaching zero in the bandgap region (Figure 3f). Moreover, a slight decrease in the bandgap is observed, accompanied by a slight shift and enhancement of the Fermi level, contributing to the improved Seebeck coefficient.

**Figure 4:**
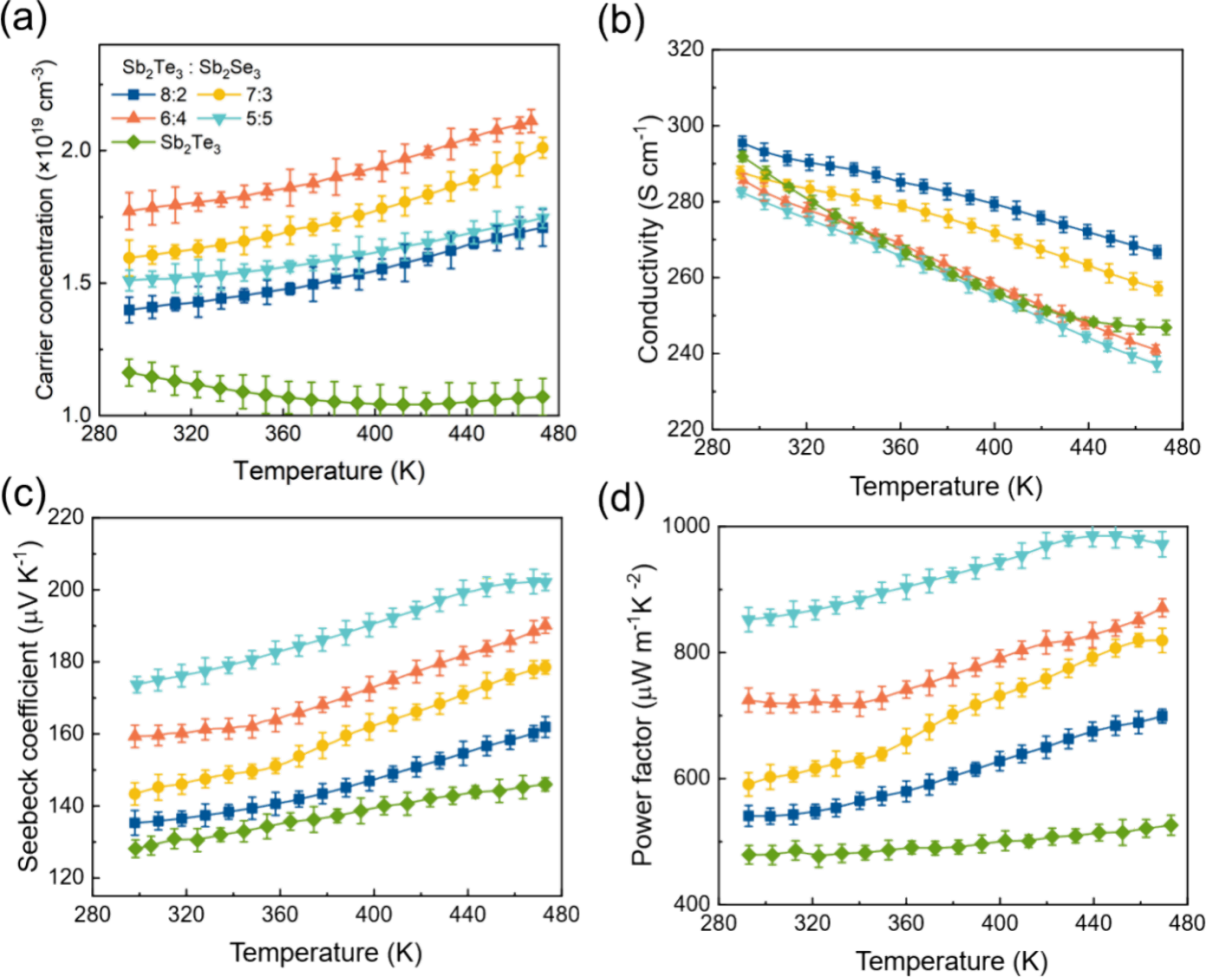
Temperature-dependent electrical transport properties of Sb_2_Te_3_–Sb_2_Se_3_ thin films: (a) carrier concentration, (b) conductivity, (c) Seebeck coefficient, and (d) power factor.

To investigate the electrical properties of Sb_2_Te_3_–Sb_2_Se_3_ nanostructures while minimizing the influence on their alloyed structure, samples with higher thickness ratios were fabricated. The temperature-dependent carrier concentration, conductivity, and Seebeck coefficient were explored, as displayed in Figure 4. The Sb_2_Te_3_–Sb_2_Se_3_ samples exhibit distinct transport behavior compared to the Sb_2_Te_3_ layer. At room temperature, the highest *n* of 1.78 × 10^19^ cm^–3^ was achieved for the sample Sb_2_Te_3_:Sb_2_Se_3_ = 6:4 (nm). The electrical conductivity of Sb_2_Te_3_–Sb_2_Se_3_ structures showed minimal variation compared to the Sb_2_Te_3_ single layer. The highest σ of 295.45 S cm^–1^ was achieved for the Sb_2_Te_3_:Sb_2_Se_3_ = 8:2 (nm) sample, closely followed by the value of 291.23 S cm^–1^ for Sb_2_Te_3_. All thin films exhibited positive *S* values, confirming their p-type semiconducting nature. A Seebeck coefficient of 174 μV K^–1^ was obtained at room temperature, representing a 35% enhancement compared to the Sb_2_Te_3_ layer. This significant increase in the Seebeck coefficient for the Sb_2_Te_3_–Sb_2_Se_3_ superlattice layers resulted in an exceptional power factor reaching up to 852 μW m^–1^ K^–2^ at room temperature.

**Figure 5:**
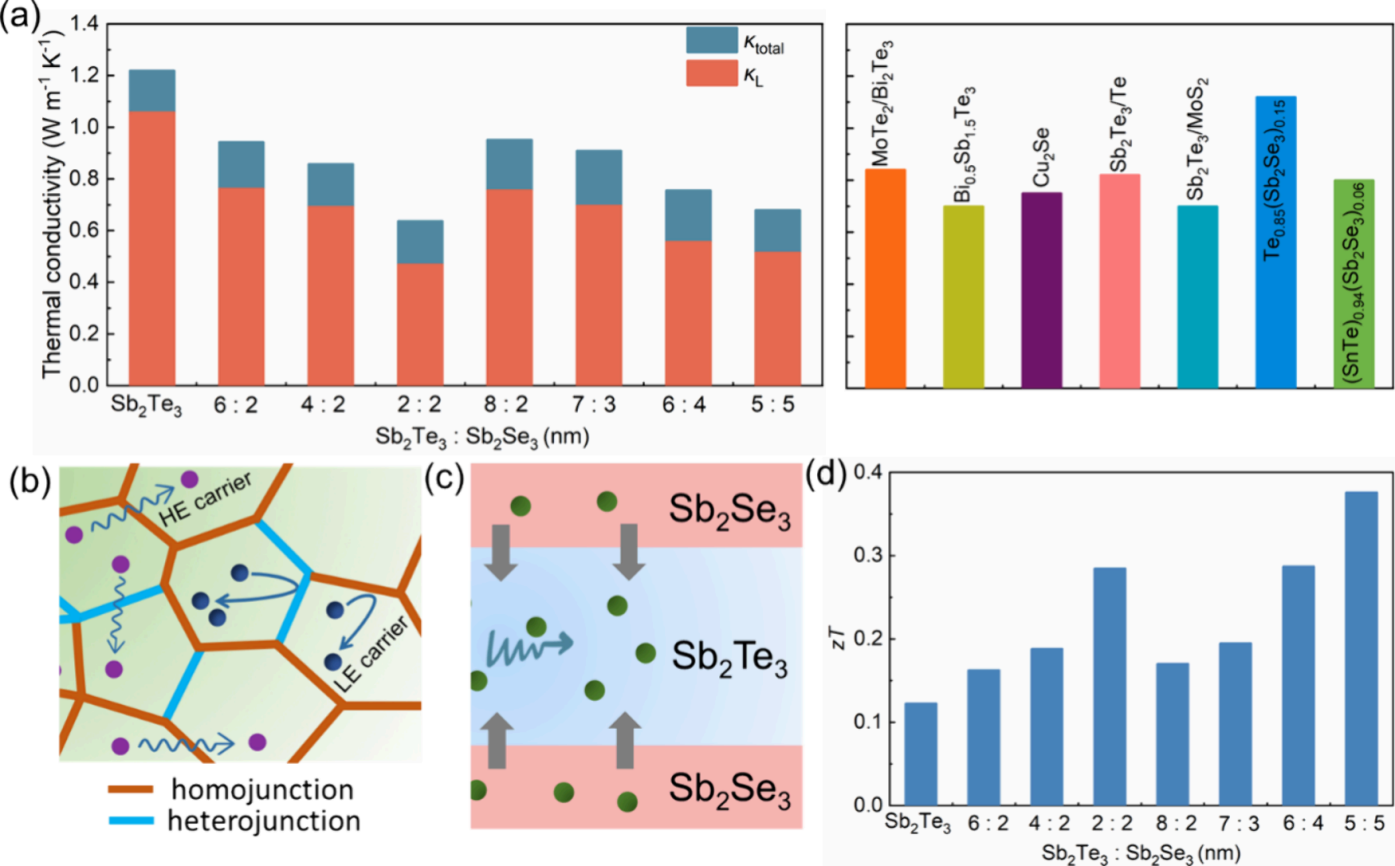
(a) Total thermal conductivity and lattice thermal conductivity of Sb_2_Te_3_–Sb_2_Se_3_ thin films and the comparison with reported data at room temperature.^10,31−37^ The models of (b) alloy and (c) superlattice structures in the thin films. (d) *zT* values for various samples at room temperature.

The thermal conductivity of Sb_2_Te_3_–Sb_2_Se_3_ films was evaluated at room temperature using the 3-ω technique (Figure 5a). At room temperature, the Sb_2_Te_3_–Sb_2_Se_3_ layers exhibited a lower total thermal conductivity (κ_Total_) compared to pure Sb_2_Te_3_ layers. The sample with a composition ratio of Sb_2_Te_3_:Sb_2_Se_3_ = 2:2 (nm) demonstrated an impressive minimum thermal conductivity of 0.62 W m^–1^ K^–1^, which is almost 2 times lower than the total thermal conductivity of Sb_2_Te_3_ (1.23 W m^–1^ K^–1^). The obtained thermal conductivity is comparable to that of multilayered structures such as Sb_2_Te_3_–MoS_2_ but lower than alloys like Te_0.85_(Sb_2_Se_3_)_0.06_ or bulk Sb_2_Te_3_ or Sb_2_Se_3_ (Table S4 in the Supporting Information), indicating the significant effectiveness of the alloyed structure and multilayer period in reducing thermal conductivity.^10,31−37^

According to Wiedemann–Franz law, the electronic (κ_E_) and lattice (κ_L_) thermal conductivities can be carried out by the following formulas:
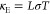
1
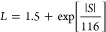
2
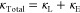
3where *L* is the Lorenz number, *T* is the temperature, and *S* is the Seebeck coefficient. The calculated κ_L_ is also shown in Figure 5a. In the Sb_2_Te_3_–Sb_2_Se_3_ system, lattice thermal conductivity primarily contributes to the total thermal conductivity. Generally, increasing the interface density of thin films decreases κ_L_, which adequately explains the overall change trend of κ_L_.^10^ Interestingly, the lattice thermal conductivity of the sample with a composition ratio of Sb_2_Te_3_:Sb_2_Se_3_ = 2:2 (nm) (0.46 W m^–1^ K^–1^) is lower than that of the sample with a composition ratio of Sb_2_Te_3_:Sb_2_Se_3_ = 5:5 (nm) (0.51 W m^–1^ K^–1^), suggesting that additional scattering occurs in the former system. This should be attributed to the different weightings of phonon scattering mechanisms in alloyed structures and quantum confinement effects in superlattice structures, resulting in distinct impacts on thermal conductivity. In systems where the average mean free path of phonons exceeds the limiting dimension of the sample, heat transport does not follow Fourier’s law, exhibiting semiballistic phonon transport characteristics.^38,39^ This regime highlights the wave nature inherent in phonons, with the potential to sustain coherence and consequently reduce the κ.^40^ Low-dimensional nanostructures exhibit favorable TE performance due to quantum confinement effects.^41,42^ For the Sb_2_Te_3_:Sb_2_Se_3_ = 2:2 (nm) sample, the Volmer–Weber growth mode results in the formation of structural features such as alloy compositions, point defects, or phase boundaries as well as superlattice structures. These structural characteristics collectively contribute to enhanced phonon scattering, consequently reducing the thermal conductivity (Figure 5b,c). In comparison, the Sb_2_Te_3_:Sb_2_Se_3_ = 5:5 (nm) sample exhibits a more ordered structure, and the quantum confinement effect is the primary factor responsible for decreasing the thermal conductivity. The rational design of the work function difference will lead to facilitate directional hole injection into the coherent region through modulation doping. To establish charge equilibrium at the interface, carrier redistribution takes place, leading to band bending. Our theoretical simulations provided strong confirmation of the results (see Tables S5–S7 in the Supporting Information). Phonons with mid- to long-wavelengths scattering are enhanced at the heterojunction interfaces, which is considerably more efficient than scattering at normal grain boundaries.^22,26,43^ Consequently, the m* increases and the thermal conductivity decreases simultaneously, leading to a high *zT* value of 0.38 for the sample Sb_2_Te_3_:Sb_2_Se_3_ = 5:5 (nm).

## 3 Conclusions

In conclusion, we designed Sb_2_Te_3_–Sb_2_Se_3_ nanostructured thin films by using thermal ALD. The transport properties, including the carrier concentration, electrical conductivity, Seebeck coefficient, and thermal conductivity, were evaluated. In the Sb_2_Te_3_:Sb_2_Se_3_ = 2:2 (nm) system, Volmer–Weber growth introduced alloy and defect structures that reduce the carrier concentration and Hall mobility. Through meticulous optimization of the sublayer thickness of Sb_2_Se_3_, a notable improvement was achieved with a high Seebeck coefficient of 174 μV K^–1^, demonstrating a 35% enhancement compared to the pure Sb_2_Te_3_ layer. As a result of the increase in *S* and σ for the Sb_2_Te_3_–Sb_2_Se_3_ systems, a high power factor reaches up to 852 μW m^–1^ K^–2^ for the sample Sb_2_Te_3_:Sb_2_Se_3_ = 5:5 (nm). The results indicated that the relative contributions of phonon scattering mechanisms and quantum confinement effects could significantly influence the overall thermoelectric performance of nanostructured thin films. This study provides valuable insights into the design and fabrication of high-performance chalcogenides via the ALD technique.

## 4 Experimental Section

### Fabrication of Sb_2_Te_3_ and Sb_2_Se_3_ Thin Films

The Sb_2_Te_3_ and Sb_2_Se_3_ thin films were grown using a thermal ALD reactor (Veeco Savannah S100) at 80 and 110 °C, respectively. SbCl_3_ and (Et_3_Si)_2_Te were used for Sb_2_Te_3_ films, while Se(SnMe_3_)_2_ and SbCl_3_ were used for Sb_2_Se_3_ synthesis. During the ALD process, the SbCl_3_, (Et_3_Si)_2_Te, and Se(SnMe_3_)_2_ were kept at 60, 77, and 60 °C, respectively. High-purity N_2_ was used as the carrier gas, and the chamber was kept at a flow rate of 10 sccm during the reaction process. The optimized pulse and purge times for one ALD deposition (precursor 1/N_2_/precursor 2/N_2_) were 1/10/1/10 s. The detailed ALD process is shown in Table S1 (Supporting Information). The sample identifier Sb_2_Te_3_:Sb_2_Se_3_ = 2:2 (nm) indicates that the individual thicknesses of Sb_2_Te_3_ and Sb_2_Se_3_ are both 2 nm. The total thickness for all samples is approximately 100 nm.

### Characterization of Morphology, Electrical, and Thermal Properties

The thickness of thin films was measured using X-ray reflectometry (X’Pert MRD PRO). Gracing incident X-ray diffraction (GID) was performed using a custom-made laboratory setup with a Mo Kα source (λ = 0.713 Å). We aligned the surface of the films at an angle of 0.5° with respect to the incident X-ray beam. The samples were then rotated about their surface normal, and diffraction images for scattering angles up to 40° were recorded using a 2D pixel detector. The detector images were transformed into reciprocal space, and the diffracted intensity was regrouped into the scattering angle (2θ) and the polar angle along the Laue circles (Χ) using the pyFAI package.^44^ The morphology and atomic structure of thin films were characterized by transmission electron microscopy using a double-corrected (aberration correction in scanning and bright-field mode) ThermoFisher Titan 80-300 operated at a 300 kV acceleration voltage. Thin films were deposited on SiO_2_ and Si_3_N_4_ substrates (TFA chip from Linseis company) for structural characterization and transport property measurements, respectively.^45,46^ The detailed platform measurement can be found in the Supporting Information.

### Theoretical Calculations

The initial structures were created using the fully relaxed Sb_2_Te_3_ bulk structure. Sb_2_Te_3_ consists of three rhombohedral stacked layers each consisting of five atomic layers with a space group of *R*3̅*m*. The heterostructure systems were made by replacing the Te with Se within one layer to form different mixing ratios of Sb_2_Te_3_/Sb_2_Se_3_ layers. Later, the effect of precursor mixing was also studied by Se–Te substitutions within one layer in the 2:2 nm system (Table S2, Supporting Information). Additionally, we also investigated Se_Te_ defects by replacing a single Te atom with Se. For creating various concentrations, 1 × 1 × 1, 2 × 2 × 1, and 3 × 3 × 1 supercells of the hexagonal unit cell were considered. The structures were relaxed until the forces were below 1 meV/Å. The electronic structures were obtained using density functional theory (DFT) with the Perdew–Burke–Ernzerhof (PBE)^47^ exchange-correlation functional as implemented in FHI-aims.^48^ It is well known that hybrid functionals such as HSE06 can lead to an opening of the bandgap,^49^ correcting (sometimes “over”-correcting) the underestimation by (semi)local functionals. On the other hand, the purpose of this study is to understand the effects of structures and interface variations on the thermoelectric coefficients. It has been shown that (semi)local functionals such as PBE can produce satisfactory results.^50,51^ Scalar relativistic corrections (ZORA), spin–orbit coupling (SOC) effects, and nonlocal many-body dispersions were included in the computations as described in the references.^52,53^ Subsequently, transport coefficients were calculated based on the Boltzmann transport equation within the constant–relaxation–time approximation employing BoltzTrap2.^54^

## Supporting Information Available

Additional characterizations such as out-of-plane XRD patterns; EDS mapping for elemental distribution; detailed DFT simulations of defect models, lattice mismatch, and interfacial charge transfer; and transport property measurement platform (PDF)
