# Supplementary material for: Interfacial Distortion of Sb2Te3–Sb2Se3 Multilayers via Atomic Layer Deposition for Enhanced Thermoelectric Properties
Source: ACS Nano. 2024 Jun 26;18(27):17500–8. doi: 10.1021/acsnano.3c13152 (PMC11238618; doi:10.1021/acsnano.3c13152)
Supplement: Supplementary file 1 — nn3c13152_si_001.pdf [file nn3c13152_si_001.pdf]

Supporting Information

Interfacial Distortion of Sb_2Te_3-Sb_2Se_3 Multilayers via Atomic Layer Deposition for Enhanced Thermoelectric Properties

Jun Yang [a,b], Mohammadreza Daqiqshirazi [c], Tobias Ritschel [d], Amin Bahrami [a], Sebastian Lehmann [a], Daniel Wolf [a], Wen Feng [a], Almut Pöhl [a], Jaroslav Charvot [e], Filip Bureš [e], Thomas Brumme [c], Axel Lubk [a], Jochen Geck [d], Kornelius Nielsch [a,b*]

[a] *Leibniz Institute for Solid State and Materials Research, 01069 Dresden, Germany*

[b] *Institute of Materials Science, Technische Universität Dresden, 01062 Dresden, Germany*

[c] *Chair of Theoretical Chemistry, Technische Universität Dresden, 01069 Dresden, Germany*

[d] *Institute of Solid State and Materials Physics, Technische Universität Dresden, 01069 Dresden, Germany*

[e] *Pardubice Institute of Organic Chemistry and Technology, Faculty of Chemical Technology, University of Pardubice, 53210 Pardubice, Czech Republic*

* *Corresponding author: Kornelius Nielsch (k.nielsch@ifw-dresden.de)*

Table S1. The process details of the Sb_2Te_3-Sb_2Se_3 system.

Layer	Precursor A (Heating Temp.)	Precursor B (Heating Temp.)	Growth Temp.	Sequence time (s)
Sb_2Te_3	$SbCl_3$ (60 °C)	$(Et_3Si)_2Te$ (77 °C)	80 °C	1-10-1-10
Sb_2Se_3		$Se(SnMe_3)_2$ (60 °C)	110 °C	1-10-1-10

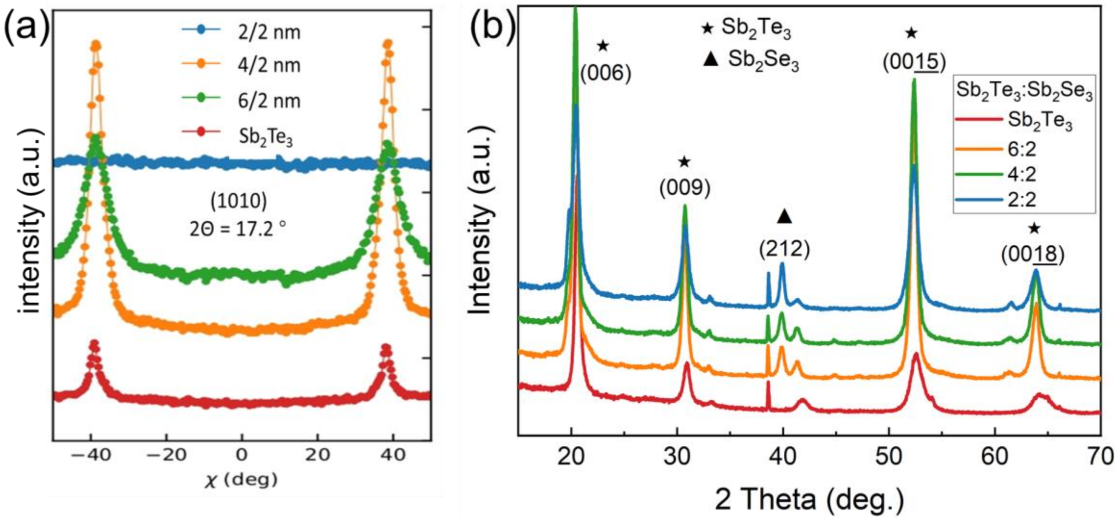

Figure S1. (a) X-Ray line scan at a constant 2θ angle of 17.2° from Figure 1 in the main article and (b) Out-of-plane XRD patterns of thin films.

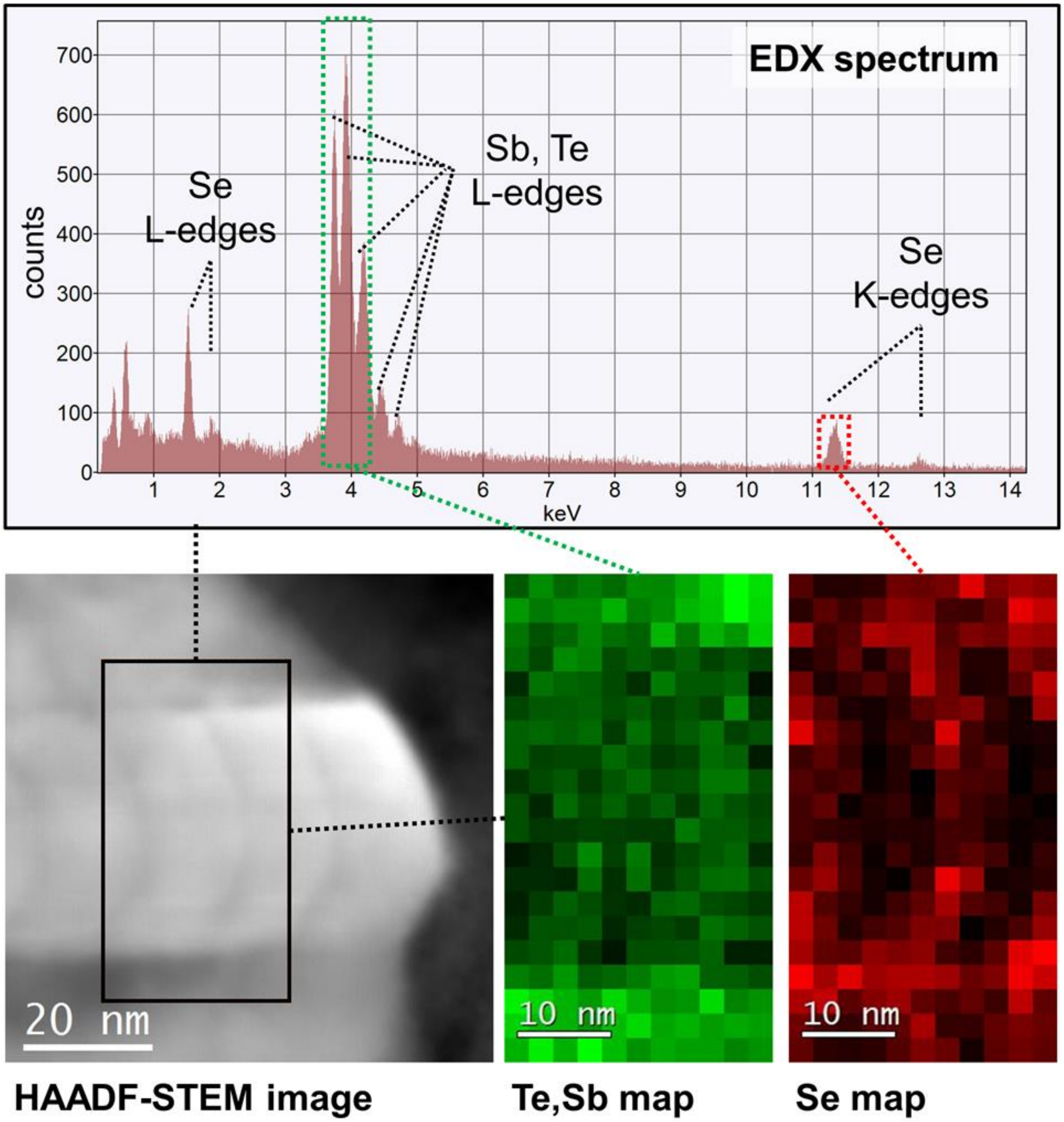

Figure S2. STEM-EDX mapping of Sb_2Te_3-Sb_2Se_3 multilayer grains. The energy dispersive X-ray (EDX) spectrum showing pronounced peaks for Sb, Te, and Se inner shell excitations is collected in the region marked as black rectangle in the HAADF-STEM image. From this region, two spectrum images at energy windows marked as green and red dashed boxes in the EDX spectrum are extracted to obtain qualitative Sb, Te (green), and Se (red) maps, respectively. Remarkably, the contrast of the Sb,Te map correlates with the bright region (higher Z contrast), whereas the Se map correlates with the dark region (lower Z contrast) in the HAADF-STEM image.

Theoretical models of transport properties affected by replacement reactions

Various atom changes or exchange systems are created for the system Sb_2Te_3:Sb_2Se_3 = 2:2 (nm). For the details of these systems, one may refer to Table S2. The systems are created by the exchange or replacement of the Telluride and Selenium atoms. System 3 is a supercell to investigate the effect of mixing inside a layer. It can be inferred from Figure S3 that the defects can alter the transport coefficients of these systems to a large extent. For example, the in-plane Seebeck coefficient of system 3 is almost two times larger than that of system 1.

Table S2. Layer elements in mixed systems of Sb_2Te_3:Sb_2Se_3 = 2:2 (nm). The atom changes or exchange is marked by a black box. For example, in system 1 a Se atom is replaced by a Te atom. Each 5-layer is shaded to help the eye.

System1	*System2*	*system3*	*system4*	*system5*	*system6*	*System7*	*system8*
Se	Se	Se	Se	Te	Te	Te	Se
Sb	Sb	Sb	Sb	Sb	Sb	Sb	Sb
Se	Se	Se	Se	Se	Se	Se	Se
Sb	Sb	Sb	Sb	Sb	Sb	Sb	Sb
Se	Se	Se	Se	Se	Se	Se	Se
Se	Se	Se	Se	Se	Se	Se	Se
Sb	Sb	Sb	Sb	Sb	Sb	Sb	Sb
Se	Se	Se	Se	Se	Se	Se	Se
Sb	Sb	Sb	Sb	Sb	Sb	Sb	Sb
Te	Te	Te-Se	Se	Se	Se	Te	Te
Te	Se	Te-Se	Te	Te	Te	Te	Te
Sb	Sb	Sb	Sb	Sb	Sb	Sb	Sb
Te	Te	Te	Te	Te	Te	Te	Te
Sb	Sb	Sb	Sb	Sb	Sb	Sb	Sb
Te	Te	Te	Te	Te	Te	Te	Te
Te	Te	Te	Te	Te	Te	Te	Te
Sb	Sb	Sb	Sb	Sb	Sb	Sb	Sb
Te	Te	Te	Te	Te	Te	Te	Te
Sb	Sb	Sb	Sb	Sb	Sb	Sb	Sb
Te	Te	Te	Se	Se	Te	Te	Se
Se	Se	Se	Se	Te	Te	Te	Se
Sb	Sb	Sb	Sb	Sb	Sb	Sb	Sb
Se	Se	Se	Se	Se	Se	Se	Se
Sb	Sb	Sb	Sb	Sb	Sb	Sb	Sb

Se	Se	Se	Se	Se	Se	Se	Se
Se	Se	Se	Se	Se	Se	Se	Se
Sb	Sb	Sb	Sb	Sb	Sb	Sb	Sb
Se	Se	Se	Se	Se	Se	Se	Se
Sb	Sb	Sb	Sb	Sb	Sb	Sb	Sb
Te	Te	Te-Se	Se	Se	Se	Te	Te
Te	Se	Te-Se	Te	Te	Te	Te	Te
Sb	Sb	Sb	Sb	Sb	Sb	Sb	Sb
Te	Te	Te	Te	Te	Te	Te	Te
Sb	Sb	Sb	Sb	Sb	Sb	Sb	Sb
Te	Te	Te	Te	Te	Te	Te	Te
Te	Te	Te	Te	Te	Te	Te	Te
Sb	Sb	Sb	Sb	Sb	Sb	Sb	Sb
Te	Te	Te	Te	Te	Te	Te	Te
Sb	Sb	Sb	Sb	Sb	Sb	Sb	Sb
Te	Te	Te	Se	Se	Te	Te	Se

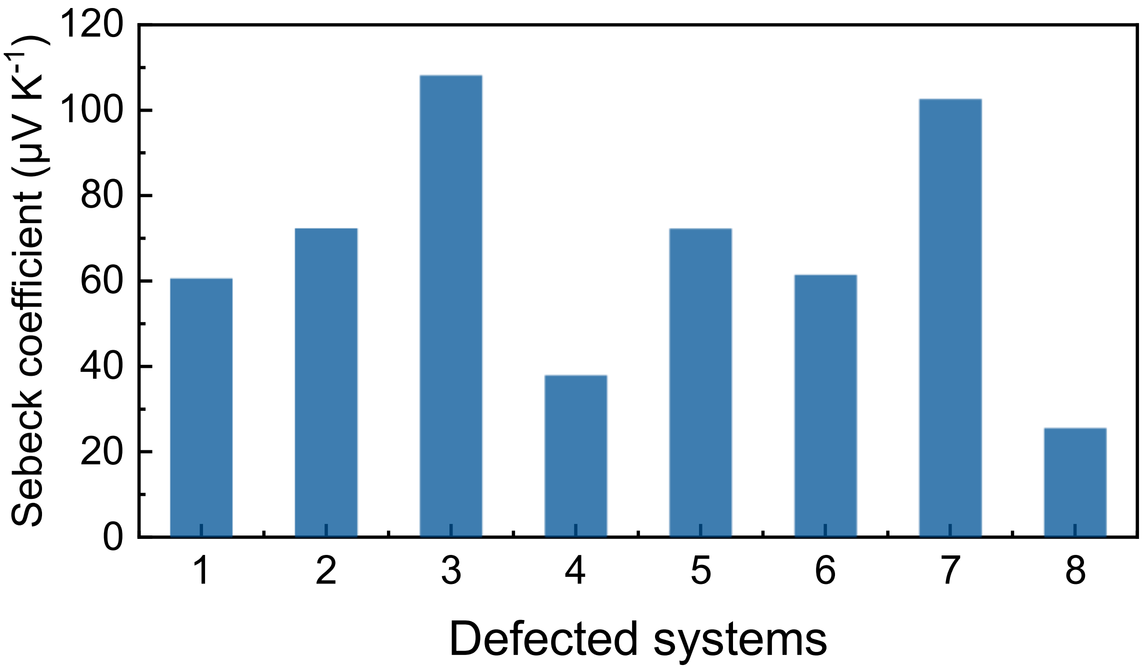

Figure S3. The in-plane Seebeck coefficient of the defected 2nm:2nm system.

Figure S4 shows the Mulliken band structure of the doped systems. In all three systems, the valence band mainly originates from Sb and Te atoms and there is some contribution from Se atoms to the conduction band.

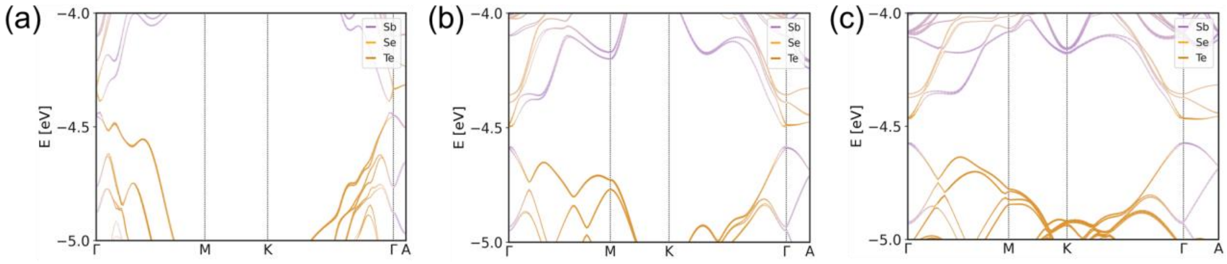

Figure S4. The Mullikan band structure of doped systems (a) 111 (b) 221 and (c) 331.

Lattice mismatch and induced interface strain

The mismatch between Sb_2Te_3 and Sb_2Se_3 could affect both the energy band structure and the in-plane transport properties. The case of 2nm:2nm is the most strained case studied in the current research. Therefore, we discuss this case as the extreme case. The relaxed lattice parameters of the systems are summarized in Table S3. The interface mismatch between these two systems (as they are relaxed separately) is 5.6%. This is the strain which is present in the Sb_2Te_3:Sb_2Se_3 = 2:2 (nm). However, we relax the system after making the heterostructures and the system of the final structure has only 1.5% strain in comparison to the relaxed lattice of a bilayer of Sb_2Te_3. The effect of these structural changes can be seen in the band structure shown in Figure S5. The band gap is not affected much by the strain and it is the states far from the Fermi energy which are mostly influenced. These factors, however, are not significant at the temperatures at which the measurements are conducted, as they are beyond a distance of $3k_bT$. The strain can alter the thermoelectric coefficient slightly due to the changes in the electronic states.

Table S3. The relaxed lattice parameters of the Sb_2Te_3:Sb_2Se_3 = 2:2 (nm) system.

Case	Lattice parameter (Å)
Two five layer Sb_2Te_3	4.27
Two five layer Sb_2Se_3	4.03
2nm:2nm	4.21

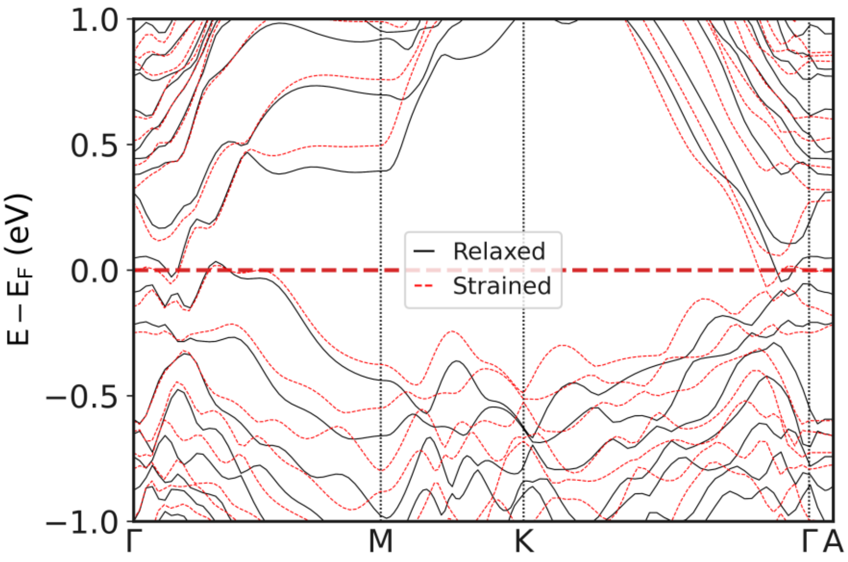

Figure S5. The band structure of Sb_2Te_3:Sb_2Se_3 = 2:2 (nm) with and without relaxation. There is a slight change in the states close to the Fermi level.

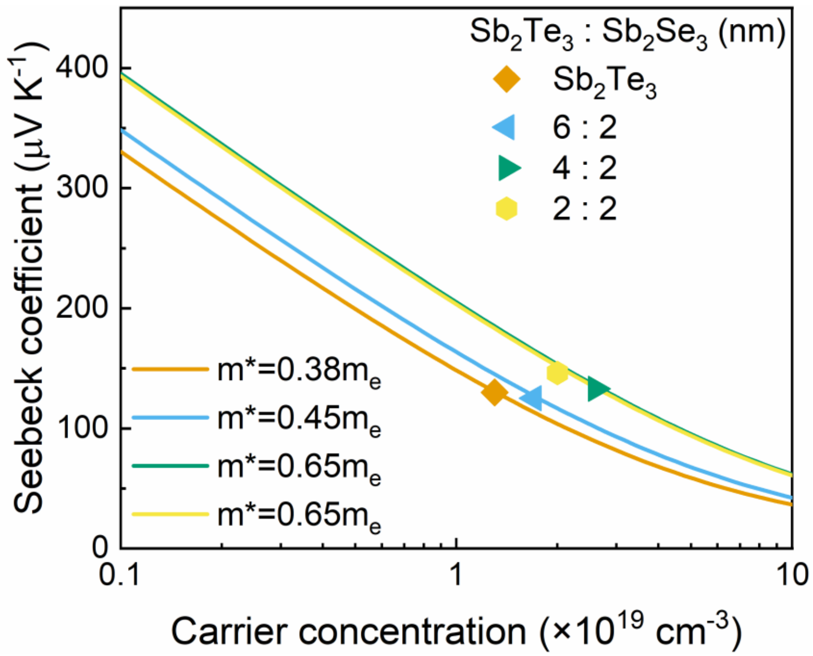

Figure S6. The Pisarenko plot of Seebeck coefficient as a function of Hall carrier concentration at room temperature (m_e is mass of a free electron).

Table S4. Room temperature transport properties of Sb_2Te_3-Sb_2Se_3 thin films and related bulk compounds.[1, 2]

Structure (ratio: nm)	n ($\times 10^{19}$ cm^{-3})	σ (S cm^{-1})	S (μV K^{-1})	PF (μW m^{-1} K^{-2})	κ (W m^{-1} K^{-1})	zT
Sb_2Te_3	1.18	295	130	499	1.23	0.12
Sb_2Te_3:Sb_2Se_3 = 6:2	1.70	327	125	512	0.94	0.16
Sb_2Te_3:Sb_2Se_3 = 4:2	2.62	304	133	539	0.85	0.19
Sb_2Te_3:Sb_2Se_3 = 2:2	2.03	283	146	605	0.62	0.28
Sb_2Te_3:Sb_2Se_3 = 8:2	1.40	295	135	541	0.95	0.17
Sb_2Te_3:Sb_2Se_3 = 7:3	1.59	296	143	590	0.90	0.19
Sb_2Te_3:Sb_2Se_3 =6:4	1.77	285	159	724	0.76	0.29
Sb_2Te_3:Sb_2Se_3 = 5:5	1.51	282	174	852	0.69	0.38
Sb_2Se_3	0.36	62	65	26	0.86	0.01
Bulk Sb_2Te_3	7.02	2200	100	2200	2.51	0.26
Bulk Sb_2Se_3	---	2.02	293	17	1.75	0.003

Theoretical models of charge transfer

Based on the Hirshfeld charge analysis, we calculated partial charges on different atoms in different systems. Tables S5-S7 show the partial charges on different atoms in bulk Sb_2Te_3, Sb_2Se_3, and the Sb_2Te_3-Sb_2Se_3 multilayered system. It can be understood from the data that a charge redistribution occurs. There are three types of atoms in the bulk Sb_2Te_3 and Sb_2Se_3: one type of antimony and two types of tellurium/ selenium based on their locations. On the other hand, there are two types of antimony and three types of tellurium/ selenium at each layer of the layered 2nm:2nm. Also, in the composite system, antimony and tellurium in telluride layer have smaller charge in comparison to bulk Sb_2Te_3.

Table S5. Partial Charges on different atoms in bulk Sb_2Te_3

Atom type/position	Partial Charge [e]
Sb	0.13
Te/interface	-0.07
Te/inside	-0.12

Table S6. Partial Charges on different atoms in bulk Sb_2Se_3

Atom type/position	Partial Charge [e]
Sb	0.21
Se/interface	-0.12
Se/inside	-0.18

Table S7. Partial Charges on different atoms in 2nm:2nm Sb_2Te_3-Sb_2Se_3 in addition to averages of charges for different layers

Atom type/position	Partial Charge [e]
Sb/Se layer, near interface	0.2
Sb/Se layer, inside	0.22
Sb/Te layer, near interface	0.11
Sb/Te layer, inside	0.11
Average Sb/Se layer	0.21
Average Sb/Te layer	0.11
Se- interface with Se layer	-0.17
Se-inside position	-0.13
Se-interface with Te layer	-0.14
Average Se	-0.15
Te-interface with Te layer	-0.06
Te- inside position	-0.11
Te- interface with Se layer	-0.04
Average Te	-0.07

Transport properties measurements

The thin films were deposited on a commercial test chip provided by Linseis company.[3, 4] A sketch of the chip is depicted in Figure S7. The samples would be prepared as follows: In the initial step, a photoresist (AZ10XT, MicroChemicals GmbH, Germany) and developer (AZ400K, MicroChemicals GmbH, Germany) were employed to create pattern alignment markers and deposit the thin film on the Linseis TFA-chip. The curing temperature was set at 110 °C for 2 minutes. Subsequently, the photoresist was removed using n-methyl pyrrolidone (NMP). All photolithography steps were carried out using a laser writer (μPG 101, Heidelberg Instruments GmbH, Germany) with a 375 nm irradiation wavelength. The transport properties were assessed over a temperature range of 293 to 473 K.

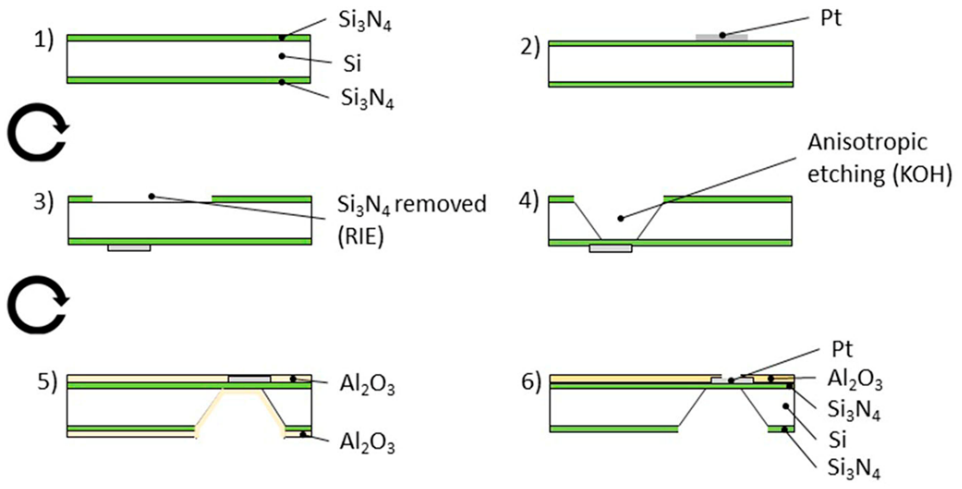

Figure S7. The sketch of the Linseis platform for thermal conductivity measurement.[4]

To ensure accurate thermal conductivity measurements, a dedicated test chip design was implemented:

1. Thin films were deposited on suspended Si_3N_4 membranes to minimize substrate heat loss.

2. A platinum rim surrounding the membranes acts as a well-defined heat sink, addressing potential size uncertainties due to fabrication variations.

3. Al_2O_3 was deposited as an additional dielectric layer to prevent shortcuts with the heater when investigating electrically conductive samples.

4. The differential method was employed to correct for substrate influence. Initially, an empty chip was measured as a reference. Subsequently, the thin film sample was deposited on the same chip for measurement. By calculating the difference between these two measurements, the thermal electric properties of the thin film could be determined, thus eliminating system errors caused by substrate effects.

5. All thermal conductivity measurements presented in this work were conducted using the 3-ω technique following the procedure outlined by Sikora et al. under quasi-steady-state conditions.[5]

By adhering to these measurement procedures, a reliable thermal conductivity value could be determined for the thin films.

References

[1] X.Y. Wang, H.J. Wang, B. Xiang, L.W. Fu, H. Zhu, D. Chai, B. Zhu, Y. Yu, N. Gao, Z.Y. Huang, F.Q. Zu, Thermoelectric Performance of Sb(2)Te(3)-Based Alloys is Improved by Introducing PN Junctions, ACS Appl Mater Interfaces, 10 (2018) 23277-23284.

[2] L.P. Hu, T.J. Zhu, X.Q. Yue, X.H. Liu, Y.G. Wang, Z.J. Xu, X.B. Zhao, Enhanced figure of merit in antimony telluride thermoelectric materials by In–Ag co-alloying for mid-temperature power generation, Acta Mater., 85 (2015) 270-278.

[3] V. Linseis, F. Völklein, H. Reith, P. Woias, K. Nielsch, Platform for in-planeZTmeasurement and Hall coefficient determination of thin films in a temperature range from 120 K up to 450 K, J. Mater. Res., 31 (2016) 3196-3204.

[4] V. Linseis, F. Volklein, H. Reith, K. Nielsch, P. Woias, Advanced platform for the in-plane ZT measurement of thin films, Rev. Sci. Instrum., 89 (2018) 015110.

[5] A. Sikora, H. Ftouni, J. Richard, C. Hébert, D. Eon, F. Omnès, O. Bourgeois, Highly sensitive thermal conductivity measurements of suspended membranes (SiN and diamond) using a 3ω-Völklein method, Rev. Sci. Instrum., 83 (2012).
